# Policies to reduce availability of tobacco products in the retail environment

**DOI:** 10.1093/eurpub/ckac129.754

**Published:** 2022-10-25

**Authors:** AE Kunst, B van den Putte, EM Veldhuizen, MAG Kuipers

**Affiliations:** 1 Department of Public and Occupational Health, Amsterdam UMC, University of Amsterdam, Amsterdam, Netherlands; 2 Amsterdam School of Communication Research, University of Amsterdam, Amsterdam, Netherlands; 3 Department of Geography and Planning, University of Amsterdam, Amsterdam, Netherlands

## Abstract

**Background:**

After implementation of a tobacco vending machine ban in 2022 and a supermarket sales ban in 2024, the Dutch government intends to further phase out tobacco sales after 2030 by prohibiting sales in petrol stations and small outlets. This study aims to understand 1) the impact of these policies on tobacco outlet availability, and 2) differences in tobacco outlet availability by area socioeconomic status (SES) in the Netherlands.

**Methods:**

Between September 2019 and June 2020, all potential tobacco retailers in four Dutch cities (Amsterdam, Eindhoven, Haarlem, and Zwolle) were visited and mapped using Global Positioning System (GPS). Expected reductions in tobacco outlet availability were calculated per policy measure. Tobacco outlet density was calculated using ESRI ArcMap version 10.4.1. The association between neighbourhood SES and tobacco outlet availability was estimated with linear and logistic regression model.

**Results:**

We identified 870 tobacco outlets and an outlet density of 6.2/10.000 capita. The potential sales bans in petrol stations and small outlets would reduce the number of outlets (resp. -7% and -43%) and the outlet density (resp. -0.4 and -2.7). In Eindhoven, Haarlem, and Zwolle, neighbourhoods with high-SES compared to low-SES were less likely to contain a tobacco outlet (OR:0.71, 95%CI:0.59-0.85) and had a lower outlet density (ß:-1.20, 95%CI:-2.20;-0.20). In Amsterdam, the associations were inverse (OR:1.22, 95%CI:1.05-1.40; ß:3.50, 95%CI:0.81;6.20).

**Conclusions:**

The availability of tobacco outlets varies within and between cities depending on the distribution of the built environment. Future tobacco control policies targeting the retail environment should focus on limiting the overall number tobacco outlets and especially small outlets, which may benefit low SES neighbourhoods in mid-sized cities most.

